# Inspissated Extract of Conium

**Published:** 1847-02

**Authors:** 


*Inspissated Extract of Conium.*—Mr. Mathews, Apothecary of this City, has sent us a specimen of the Extract of Conium, which he assures us is the veritable succus inspissatus of the plant. As this is one of the most uncertain articles in the Materia Medica, we take pleasure in directing attention to the above fact.

